# Comparing the hippocampal miRNA expression profiles of wild and domesticated Chinese tree shrews (*Tupaia belangeri chinensis*)

**DOI:** 10.1186/s12862-020-01740-2

**Published:** 2021-01-25

**Authors:** Caixia Lu, Mingxue Li, Xiaomei Sun, Na Li, Wenguang Wang, Pinfen Tong, Jiejie Dai

**Affiliations:** 1 Center of Tree Shrew Germplasm Resources, Institute of Medical Biology, Chinese Academy of Medical Science and Peking Union Medical College, Kunming, China; 2 Yunnan Key Laboratory of Vaccine Research and Development On Severe Infectious Diseases, Kunming, China

**Keywords:** miRNA-seq, Tree shrew, Domestication, Hippocampus

## Abstract

**Background:**

The domestication of tree shrews represents an important advance in the development of standardized laboratory animals. Little is known regarding the miRNA changes that accompany the transformation of wild tree shrews into domestic tree shrews.

**Results:**

By performing miRNA-seq analysis on wild and domestic tree shrews, we identified 2410 miRNAs and 30 differentially expressed miRNAs in the hippocampus during tree shrew domestication. A KEGG analysis of the differentially expressed genes showed that the differentially expressed miRNAs were associated with ECM-receptor interaction, the phosphatidylinositol signaling system, protein digestion and absorption, inositol phosphate metabolism, lysine degradation, fatty acid degradation and focal adhesion. Most of these pathways could be classified under environmental information processing, organismal systems and metabolism. The miRNAs exclusively expressed in wild and tame tree shrews GO enriched in terms of divergent functions. The miRNA-mRNA networks suggested that novel-m1388-5p and novel-m0746-5p might play regulatory roles in domestication of tree shrews. Real–time RT-PCR analysis was employed to verify the presence of these miRNAs.

**Conclusion:**

We identified a number of candidate miRNA-regulated domestication genes that may represent targets for selection during the domestication of tree shrews.

## Background

MicroRNAs (miRNAs) are endogenous, noncoding RNAs that are crucial for both the transcriptional and posttranscriptional regulation of gene expression in both the plant and animal kingdoms [1–3]. To date, many reports have indicated that miRNAs play roles in animal and plant domestication [2, 4, 5] Federica Di Palma *et.al* compared the evolution of miRNAs in five domestic species of considerable economic and biomedical importance, namely, cows, dogs, horses, pigs and rabbits, and found that the associated targets highlight the presence of several genes under artificial positive selection, suggesting the involvement of these miRNAs in the domestication process [3]. Y. Liu *et.al* reported that miRNAs have higher rates of evolution than miRNA targets during soybean domestication and improvement [5].

The Chinese tree shrew (*Tupaia belangeri chinensis*), is a squirrel-like mammal, with a low cost of maintenance and a short reproductive cycle. Sequencing and comparison of the Chinese tree shrew genome with those of 14 other species showed that the tree shrew is closest to primates [6]. This species is widely used as a potential model for biomedical research on such viruses as hepatitis C virus (HCV) [7, 8] and hepatitis B virus (HBV) [9]. Due to the high brain-to body mass ratio of this species [10, 11], it has made an important contribution to the study of nervous system disease models, including social stress [12], depression [13], aging [14–16], Alzheimer’s disease and Parkinson’s disease [17].

Previous studies have suggested that genes affecting brain and neural development are particularly targeted during the domestication process [18]. Some highly domesticated mammals such as pigs and sheep often have a size reduction of the hippocampus, indicating that the hippocampus might play a role in the development of tame behavior in domesticated animals [19, 20]. At present, tree shrews are mostly domesticated after being captured from the field environment. In domesticated tree shrews and their offspring, pups appear to have eagerly sought contact with humans. When the breeder is near, tree shrews can eat in the hands of the breeder instead of avoiding them. Whether miRNAs change in the process of domestication, to regulate the related genes leading to such phenotypic changes in tree shrews has not been determined. In the present study, we investigated the expression profiles of miRNAs in the hippocampus of tree shrews using high-throughput sequencing during the domestication process. We also constructed miRNA-mRNA regulatory networks to provide a profile that may help elucidate the mechanisms underlying tree shrew domestication.

## Results

### Overview of miRNA-Seq

**Fig. 1:**
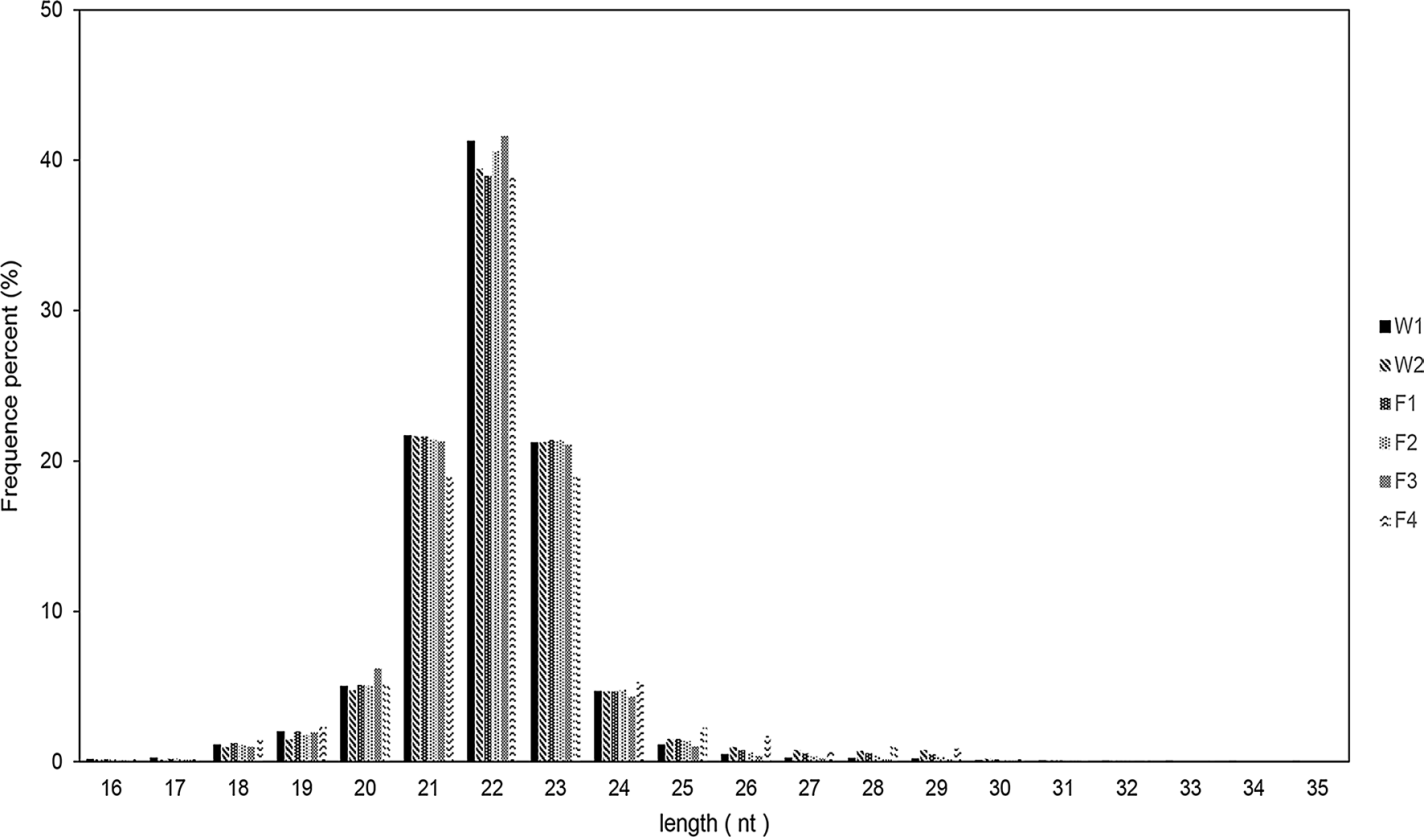
Length distribution of sequenced miRNAs. The reads with a length of 22 nt were the most abundant, followed by 21- and 23-nt reads

Using Illumina HiSeq2500 sequencing, we obtained 12,285,640 11,857,538, 10,559,041, 10,442,413 10,062,894 and 11,519,597 clean reads from the six small RNA libraries of the hippocampus tissue in two wild and four domestic Chinese tree shrews (Additional file 1: Table S1). After filtering, 12,127,859, 11,697,402, 10,415,806 10,296,956 9,928,461 and 11,291,252 high-quality clean reads were eventually obtained from the six libraries. The 18–35-nt small RNA sequences were aligned with the Chinese tree shrew genome sequence (http://asia.ensembl.org/Tupaia_belangeri/Info/Index). These mapped sequences were employed for subsequent exist miRNA identification. The unmapped sequences were compared with the known animal miRNAs in miRBase to identify the known miRNAs. The new miRNA was identified by predicting the hairpin structure combined with the reference Chinese tree shrew sequence. Through this approach, 2,410 miRNAs were detected (Additional file 2: Table S2). Among these miRNAs, 160 were exist miRNAs, 745 were known miRNAs and 1504 were new miRNAs. As shown in Fig. 1, most of the small RNAs measured approximately 22 nt in length, as observed in previous studies[21]. The number of miRNAs in all samples is shown in Additional file 2: Table S2.

### Differentially expressed (DE) miRNAs observed during tree shrew domestication

**Fig. 2:**
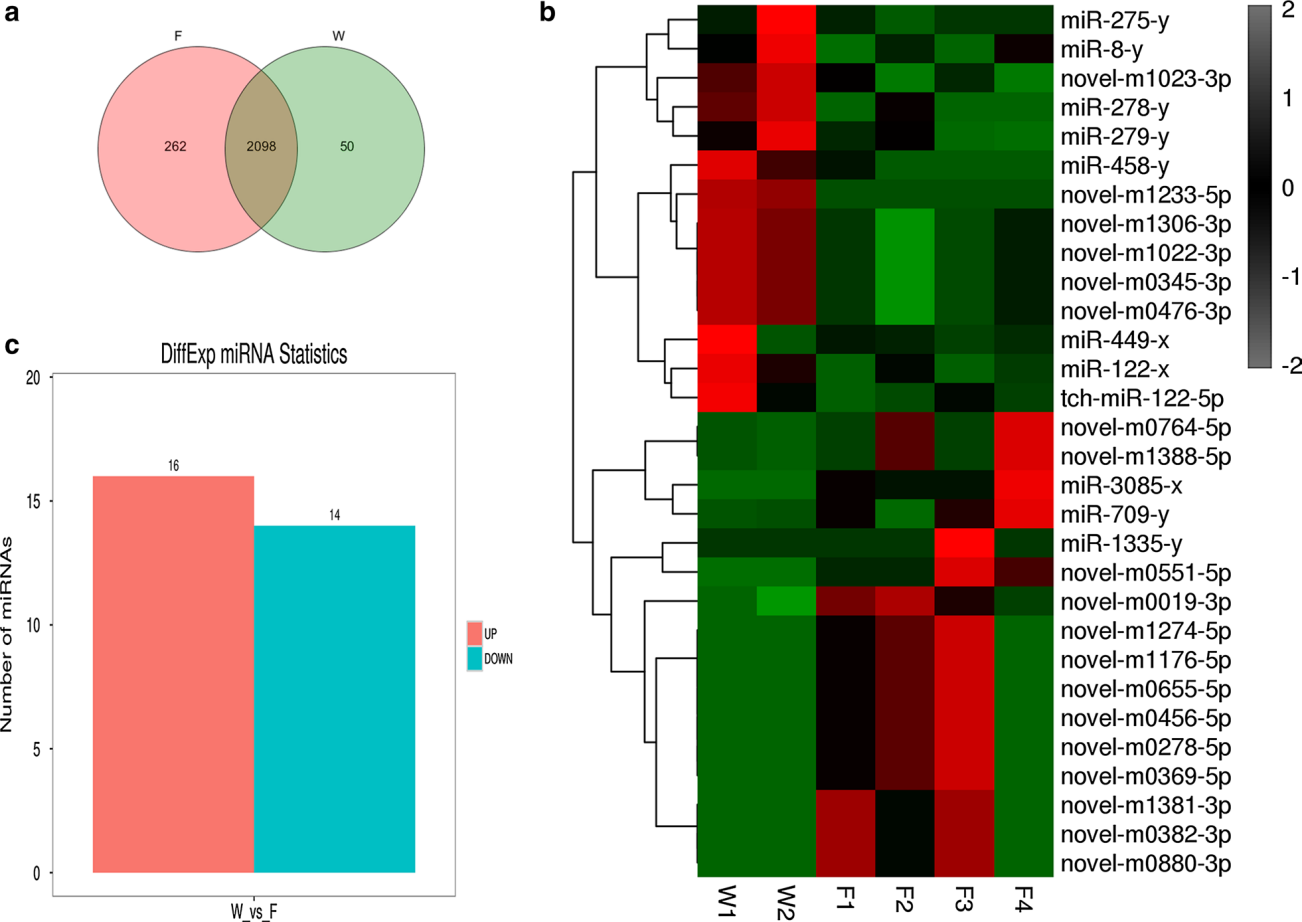
Expression profiles of differentially expressed (DE) miRNAs during tree shrew domestication. **a** Venn diagram showing the overlapping and exclusive miRNAs identified in W and F. **b** Bar plot representation of differentially expressed miRNAs in different groups. **c** Heat map of the expression profile of DE miRNAs in W and F. W represents the wild tree shrews, and F represents the domestic tree shrews

During tree shrew domestication, we detected 2,410 miRNAs in wild and domesticated Chinese tree shrews. The majority of the miRNAs annotated in wild tree shrews were also detected in the domestic tree shrews, and the reverse was also observed (Fig. 2a). In total, 50 miRNAs were expressed exclusively in the wild tree shrews, and 262 were expressed exclusively in the domestic tree shrews (Fig. 2a). Most of the specific miRNAs in F are new miRNAs, suggesting that new miRNAs have evolved in the process of domestication, and these genes may represent potential sites for domestication selection.

We also performed expression profiling to determine whether miRNAs were differentially expressed in wild and domestic tree shrews during domestication. The results showed that16 miRNAs were significantly upregulated and that14 miRNAs were significantly downregulated in W compared with F (Fig. 2b).These significant expression differences between wild and domestic tree shrews suggest that the expression of miRNA could also be a target of domestication.

### Enrichment of exclusively and differentially expressed miRNAs.

**Fig. 3:**
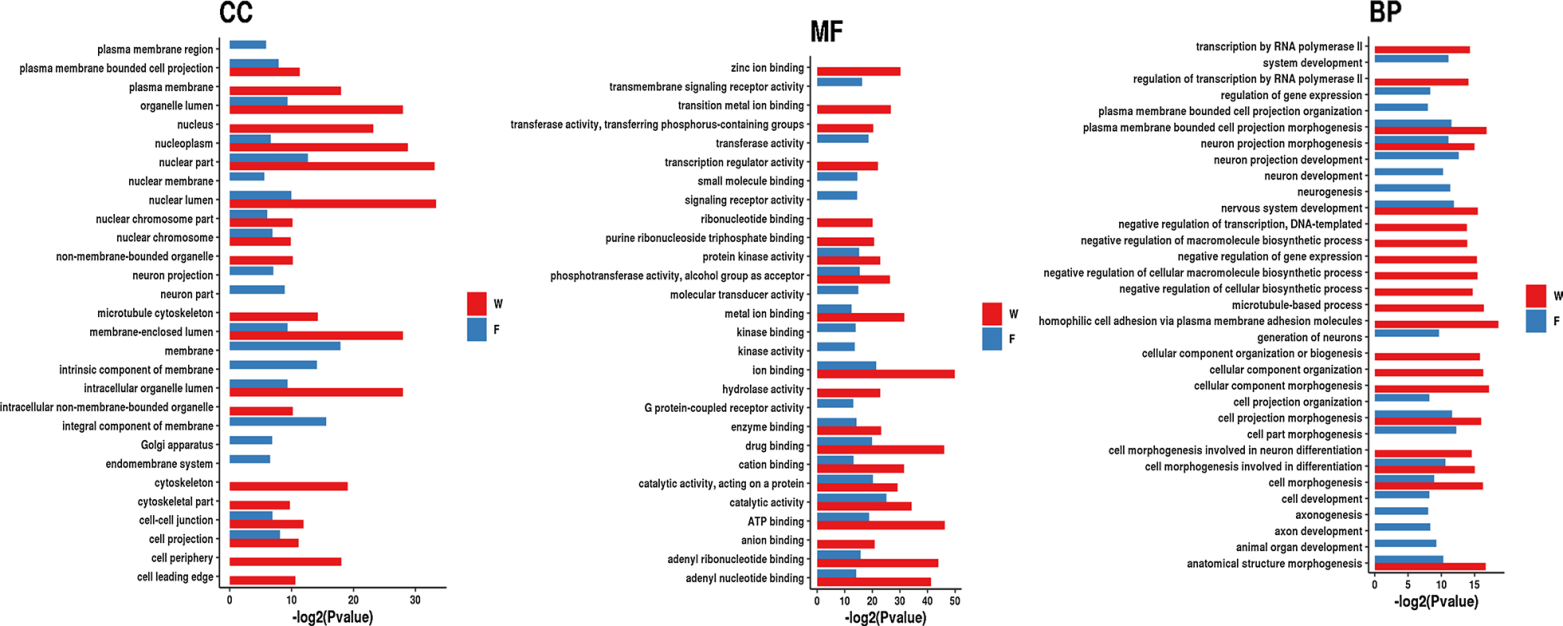
Bar plot representation of the GO terms associated with miRNAs in W and F. GO terms for the miRNAs in W and F enriched divergent functions. The length of the bar represents the statistical significance of the pathway. Red represents the W. The blue bat represents the F. W represents the wild tree shrews, and F represents the domestic tree shrews

To elucidate the possible roles played by the exclusively and differentially expressed miRNAs during domestication, enriched Gene Ontology (GO) and Kyoto Encyclopedia of Genes and Genomes (KEGG) pathways were obtained for the miRNAs in W compared with F. For exclusively expressed miRNAs, GO analyses showed that the miRNAs in the two groups were enriched in terms of divergent functions (Fig. 3). In W, 27 pathways satisfied the requirement of p < 0.05 (Additional file 3: Tables S3), however in F, only 1 pathway (the cAMP signaling pathway) satisfied the requirement of p < 0.05 (Additional file 4: Tables S4). We utilized bubble charts to represent the top 20 pathways in W and F (Additional file 5: Fig. S1). The cAMP signaling pathway was identified in both W and F (Additional file 5: Fig. S1).

**Fig. 4:**
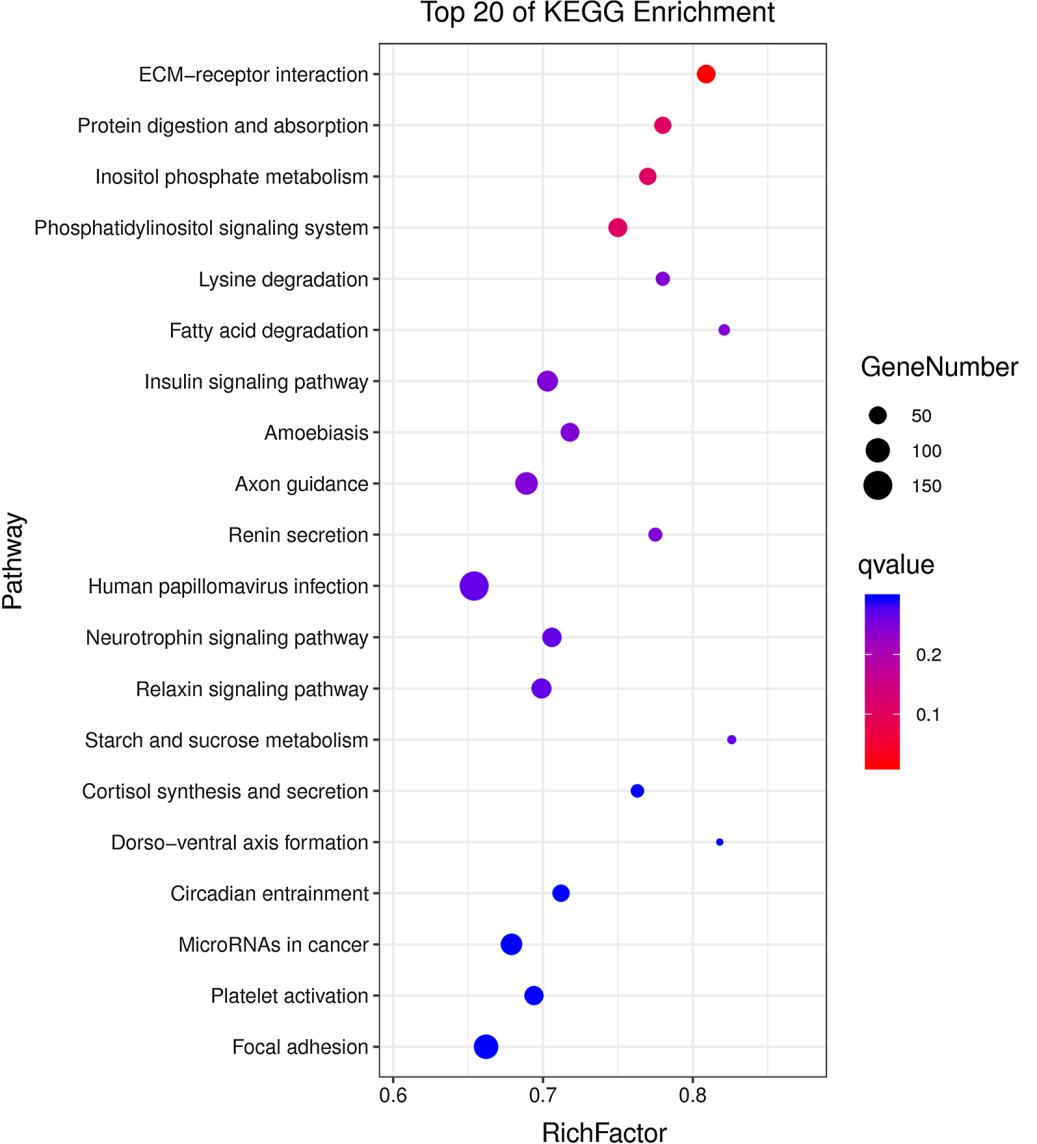
KEGG analysis of DE miRNAs in W vs F. The bubble chart shows the top 20 pathways enriched differentially expressed genes in signaling pathways. The Y-axis label represents the pathway and the X-axis label represents the rich factor (rich factor = amount of differentially expressed genes enriched in the pathway/amount of all genes in background gene set). The color and size of the bubble represent enrichment significance and the number of differentially expressed genes enriched in the pathway, respectively

For differentially expressed (DE) miRNAs, GO analyses showed that 155 terms in cellular component (CC), 160 terms in molecular function (MF) and 400 terms in biological process (BP) satisfied the requirement of p < 0.05 (Additional file 6: Tables S5).These results showed that there were significant differences in CC, MF and BP during domestication. KEGG analyses indicated that 38 pathways satisfied the requirement of p < 0.05 (Additional file 7: Tables S6), and we utilized bubble charts to represent the top 20 pathways in W compared with F (Fig. 4). As shown in Fig. 4, ECM-receptor interaction, the phosphatidylinositol signaling system, protein digestion and absorption, inositol phosphate metabolism, lysine degradation, fatty acid degradation and focal adhesion were identified in W compared with F. These results suggested that the miRNAs involved in these pathways may be related to tree shrew domestication. Furthermore, most of the pathways were classified under environmental information processing, organismal systems and metabolism (Additional file 6: Table S6).

### MiRNA-mRNA coexpression during tree shrew domestication

**Fig. 5:**
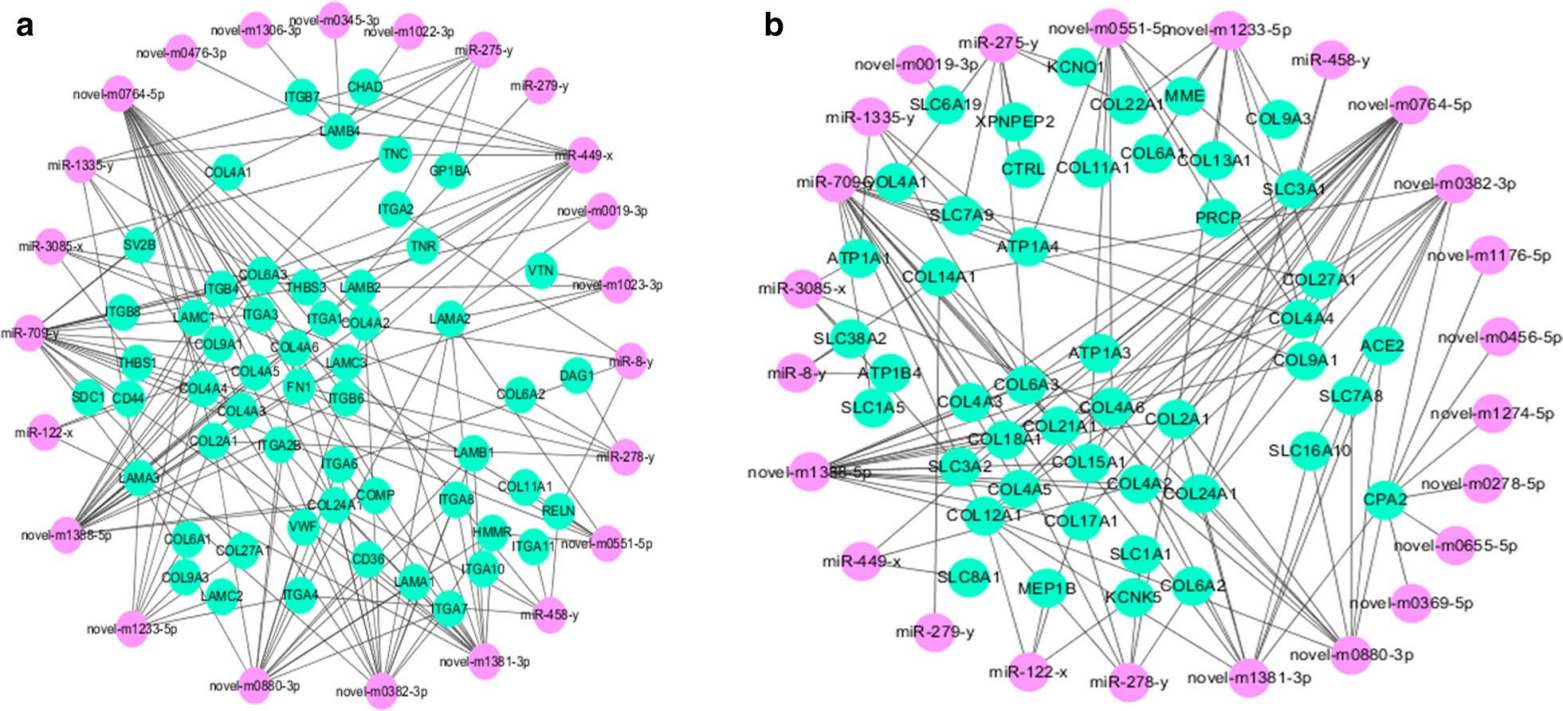
Co-expression network analysis of the miRNA-mRNA. **a** MiRNA-mRNA coexpression in the ECM-receptor interaction. **b** MiRNA-mRNA coexpression in protein digestion and absorption. Pink circles represent the miRNAs. Blackish green circles represent genes

MicroRNAs can serve as regulators of posttranscriptional gene expression by inhibiting or degrading target mRNAs [22], therefore, we constructed a miRNA-mRNA network based on ECM-receptor interaction and protein digestion and absorption. In the ECM-receptor interaction, novel-m1388-5p and novel-m0746-5p were predicted to combine with 17 of the same target genes (I.e., LAMC3, LAMC1, LAMB2, ITGA1, ITGA3, ITGA2B, FN1,COMP, COL9A1, COL6A3, COL4A6, COL4A5,COL4A4, COL4A3, COL4A2, COL2A1, and COL24A1), novel-m1388-5p was predicted to combine with ITGB6 and ITGB4, and novel-m0746-5p predicated to combine with ITGA4 and ITGA6 (Fig. 5a). In protein digestion and absorption, novel-m1388-5p and novel-m0746-5p were predicted to combine with 16 of the same target genes (8 genes were the same as those in the ECM-receptor interaction, namely, COL9A1, COL6A3, COL4A6, COL4A4, COL4A3, COL4A2, COL2A1, and COL24A1), as shown in Fig. 4b. These predicted target genes were mostly associated with collagen genes, and the genes in the two pathways were regulated by the same two miRNAs (Fig. 5). This network suggested that novel-m1388-5p and novel-m0746-5p might play regulatory roles in tree shrew domestication.

### Validation of miRNAs by quantitative RT-PCR analysis

**Fig. 6:**
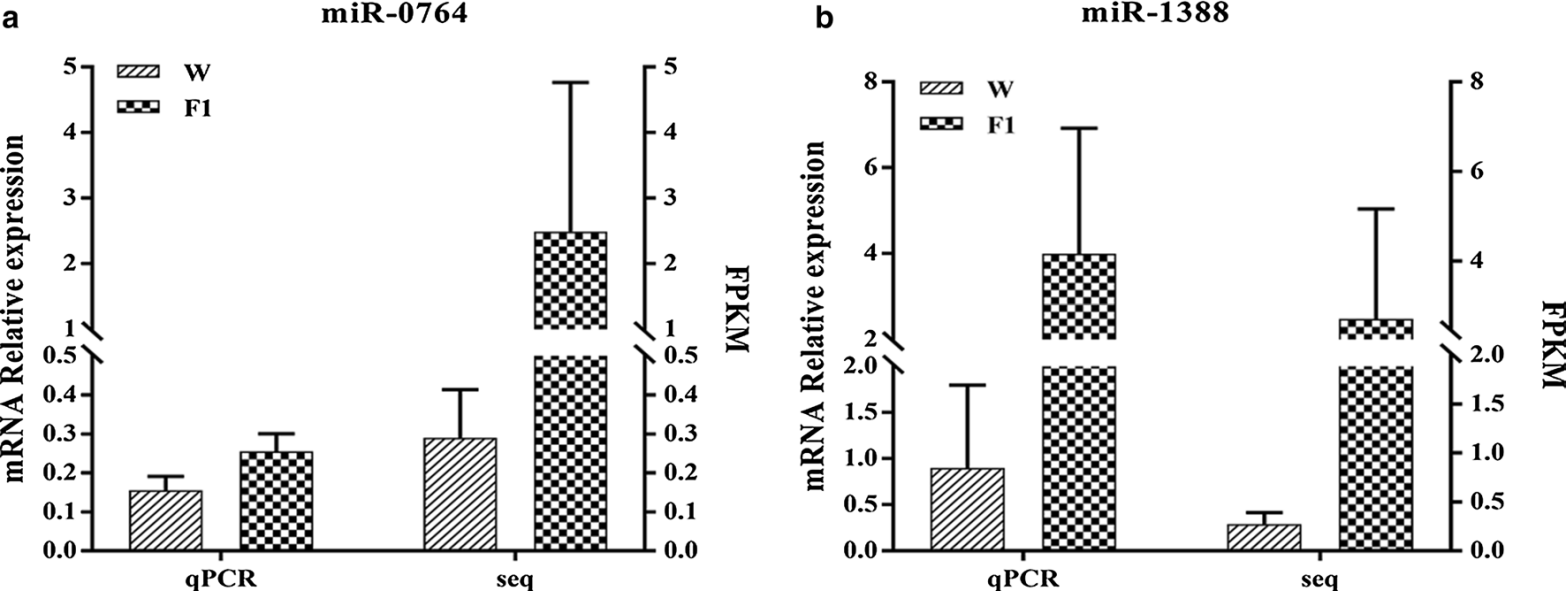
RT-PCR validation of the selected miRNAs. **a** Novel-m0746-5p. **b** Novel-m1388-5p

Next we performed qRT-PCR validation of two differentially expressed miRNAs, namely, novel-m1388-5p and novel-m0746-5p. The two miRNAs were both upregulated miRNAs, and predicted to combine with most of the same target genes (Fig. 5). The expression level of the two miRNAs was normalized to U6. As anticipated, the results of the qRT-PCR analysis (Fig. 6) were consistent with the miRNA-Seq results.

## Discussion

In this study, using high-throughput sequencing, we identified 2410 miRNAs in the hippocampus during tree shrew domestication. We observed that most of the miRNAs measured approximately 22 nt in length, as also reported in our previous studies [21]. In the total identified miRNAs, 262 miRNAs were expressed exclusively in the domestic tree shrews (Fig. 2a), and the miRNAs were mostly identified as new miRNAs. The results indicated that these new miRNAs might evolve during the process of domestication, and their target genes may represent potential sites for domestication selection. However, further research is warranted to determine the exact functions of these genes in tree shrew domestication. Gene enrichment analyses of exclusively expressed miRNAs in W and F demonstrated that miRNAs in the two groups were enriched in terms of divergent functions (Fig. 3), suggesting that these miRNAs played different roles in the two groups and might be associated with the various manifestations of domestication.

We observed 30 differentially expressed hippocampal miRNAs between wild-type and tame tree shrews (Fig. 2a). The KEGG analysis of the differentially expressed genes showed that the differentially expressed miRNAs were associated with ECM-receptor interaction, the phosphatidylinositol signaling system, protein digestion and absorption, inositol phosphate metabolism, lysine degradation, fatty acid degradation and focal adhesion. Furthermore most of the pathways were classified into environmental information processing, organismal systems and metabolism. The tame tree shrews have developed physiological features adapted to the laboratory environment as they no longer fear humans during domestication, whereas wild tree shrews do not have such features. It was reported that neurogenesis in the temporal hippocampus might contribute to the regulation of these emotional functions [20, 23, 24]. These findings suggest that the earliest stages of domestication may involve adaptation to captive environment and that exclusively and differentially expressed miRNAs may play regulatory roles in the domestication process.

Furthermore, we constructed miRNA-mRNA networks that significantly participated in ECM-receptor interaction and protein digestion and absorption. The extracellular matrix–receptor (ECM-receptor) consists of a complex mixture of structural and functional macromolecules and plays an important role in tissue and organ morphogenesis and in the maintenance of cell and tissue structure and function. Specific interactions between cells and the ECM are mediated by transmembrane molecules. These interactions lead to direct or indirect control of such cellular activities as adhesion, migration, differentiation, proliferation, and apoptosis. These interactions may be classified under environmental information processing [25]. We observed that novel-m1388-5p and novel-m0746-5p were predicted to combine with 17 and 16 of the same target genes in ECM-receptor interaction and protein digestion and absorption, respectively (Fig. 5). Furthermore, novel-m1388-5p and novel-m0746-5p were combined with 8 same genes (i.e., COL9A1, COL6A3, COL4A6, COL4A4, COL4A3, COL4A2, COL2A1, and COL24A1) in both pathways (Fig. 5b). These predicted target genes were mostly associated with collagen genes. The results of a study in *Caenorhabditis elegans* indicated that the col-150, col-121 and dpy-5 genes played important roles in the regulation of reproduction in worms, suggesting that col-131 might be an essential gene in the ECM-receptor interaction pathway and may regulate other collagen genes including col-150, col-121 and dpy-5. These collagen genes may also be related to the formation of the vulva epidermis of *Caenorhabditis elegans *[26]. Another study observed that COL6A2 (collagen, VI type, α2) played a major role in cell adhesion and affected the secretion of testicular Leydig cells [27]. Changchun Li *et.al* reported that five genes (CTNNA2, ITGB1, ITGA4, LIMS1 and COL6A2) and two miRNAs (miR218-5p and miR-221-5p) were involved in seven major categories that are closely related to spermatogenesis[28]. ITGB1 was also determined to be associated with embryonic testicular cord formation [29]. In tree shrew domestication, we observed that some male animals had penile ptosis and the pregnancy rate was lower than that of tree shrews captured from the field environment, which was consistent with the phenomena observed by other researchers. Yu Fa rong *et.al* found stress symptoms such as loss of appetite, testicular atrophy, prolapsed penis and reduced sexual behavior in tree shrews raised in small cages [30].The living conditions of tree shrews raised in the laboratory have been modified, and their dietary structure has also undergone certain changes, which may lead to changes in their behavior and psychology. Our results indicated that the differentially expressed miRNAs involved in the ECM-receptor interaction pathway were mostly related to collagen genes, which suggests that these miRNAs may be associated with penile function and reproduction. However, further research warranted to determine the exact functions of these pathways in domestication, and to increase the sample size to further verify whether the observed behavioral and psychological phenomena are related to changes in microRNA expression in the hippocampus. From the present study, we should optimize the size of the feeding cage, enrich the feed food structure, and simulate the diet structure and growth conditions that exist in the wild as much as possible.

## Conclusion

We identified a number of candidate miRNA-regulated domestication genes that may underlie some of the phenotypic traits that distinguish tame tree shrews from wild tree shrews. To draw more general conclusions regarding the outcome of the interactions between wild and tame tree shrews, further research on multiple species and individuals with longer domestication times is warranted.

## Methods

### Animals and tissue collection

Six male Chinese tree shrews aged 6–12 months and weighing 120–150 g, specifically two wild (W1 and W2) and four domestic (F1, F2, F3, and F4) Chinese tree shrews were chosen. The wild Chinese tree shrews were captured from the wild (Lufeng, Chuxiong, Yunnan Province), and the domestic Chinese tree shrews were born and bred in the laboratory. Among the four domestic tree shrews, there are two first filial and two second filial generations. The Chinese tree shrews were provided by the Institute of Medical Biology, Chinese Academy of Medical Science and Peking Union Medical College, Kunming, PR China. Tree shrews were found to be healthy, consistent with the group standards of tree shrews (T/CALAS 08–2017 and T/CALAS 09–2017), without visible signs of tumorigenesis or disease. The Chinese tree shrews were sacrificed by intraperitoneal injection of barbital sodium (100 mg/kg). Next hippocampal tissues were immediately removed, placed in RNAlater (Ambion, AM7020) and stored at -80 °C until use. All animal experiments were approved by the institutional Ethics Committee, and all procedures were performed according to ethics standards and practices.

### MiRNA extraction and RNA-Seq

Total RNA was extracted from the hippocampal tissues of each Chinese tree shrew using a miRNeasy Mini Kit (Cat#217004, QIAGEN, GmBH, Germany) according to the manufacturer’s protocol. The quality of the RNA was measured on an Agilent 2100 Bioanalyzer (Agilent Technologies).

For miRNA-seq, after isolating the total RNA, RNA molecules in a size range of 18–30 nt were enriched by polyacrylamide gel electrophoresis (PAGE). Next, 3′ adapters were added, and 36–44 nt RNAs were enriched. After that step 5′ adapters were ligated to the RNAs as well. The ligation products were reverse-transcribed by PCR amplification, and 140–160 bp PCR products were enriched to generate a cDNA library. MiRNA sequencing was performed using the Illumina HiSeq 2500 platform by Gene Denovo Biotechnology Co., Ltd. (Guangzhou, China).

### MiRNA sequencing analysis

Using the raw FASTQ files, we removed the low-quality sequences to acquire the clean reads. Next the clean reads were mapped using the GenBank database (release 209.0) and the Rfam database (version 11.0; http://rfam.sanger.ac.uk/) to identify and remove rRNA, scRNA, sonRNA, snRNA and tRNA. Clean reads were aligned with a reference genome to remove repeat sequences. Afterwards, the clean reads were initially searched against miRBase (version 21; http:// www.mirbase.org/) to identify known *Tupaia belangeri chinensis* miRNAs (existing miRNAs). For some miRNA sequences still not included in the miRBase database, the miRNAs were aligned with other species to identify known miRNAs. All the unannotated tags were aligned with the reference genome. According to their genome positions and hairpin structures predicted by Mireap_v0.2 software, novel miRNA candidates were identified.

### Differentially expressed miRNA (DE miRNA) analysis

The procedures to identify differentially expressed miRNAs across the two groups were as previously described [31]. We considered miRNAs with FC ≥ 2 and *P* values < 0.05 in comparisons of samples to be significantly differentially expressed miRNAs. Volcano plots were generated using gglpot2 in R. The heat map and Venn diagrams were generated using online tools (https://www.omicshare.com/tools/Home/Soft/heatmap and.

www.omicshare.com/tools/Home/Soft/Venn).

### Prediction of potential miRNA target genes

The potential target genes of the miRNAs were predicted with three different miRNA target prediction algorithms: Miranda (v3.3a), TargetScan (version: 7.0) and RNAhybrid (v2.1.2) + slight (v6.01). The intersection of the results was considered to include more credible predicted miRNA target genes.

### GO and KEGG pathway analyses

Gene Ontology (GO) is a standardized vocabulary that the Candida Genome Database (CGD) and other groups use to describe the functions of gene products across numerous species [32, 33]. The GO categories were derived from the GO database (http://www.geneontology.org), which is comprised of a structured set of terms that includes three domains: Molecular Function, Biological Processes, and Cellular Components [32, 34]. Kyoto Encyclopedia of Genes and Genomes (KEGG) is a database of biological systems that integrates genomic chemical and systemic functional information, which provides a knowledge base for understanding the biological roles of differentially expressed genes through PATHWAY mapping [35].

The GO and KEGG pathway analyses were performed as described in previous reports [36]*.* In briefly, the potential target genes for differentially expressed miRNAs were mapped to GO terms in the GO database (http://www.geneontology.org/), where FDR ≤ 0.05 was taken as the threshold, and the gene number of each GO term was calculated. The KEGG pathway analysis of differentially expressed genes was performed using KOBAS database (http://kobas.cbi.pku.edu.cn/home.do). Pathways with *P* values < 0.05 were considered to be significant. Bubble charts were generated using gglpot2 in R.

### Construction of the miRNAs-mRNAs network

We identified the interactions between miRNAs and mRNAs by constructing coexpression networks. We selected genes involved with ECM-receptor interaction and protein digestion and absorption (P < 0.05, and significantly differentially expressed in W vs F), and used Cytoscape (V3.2.0) to establish a miRNA-mRNA pathway regulation network.

### Data access

The datasets generated and analysed during the current study are available in the NCBI SRA repository (https://www.ncbi.nlm.nih.gov/sra/, Accession: PRJNA399798).

### Validation of miRNAs by quantitative RT-PCR analysis

For further validation, we selected 2 differentially expressed miRNAs for qRT-PCR analysis using a Mir-X miRNA First-strand synthesis and TB Green® Premix Ex Taq™ II (Tli RNaseH Plus) Kit (TAKAR, JAP). Twelve Chinese tree shrews were used (five wild and eight domestic), the miRNA was isolated using RNAiso Plus (TAKAR, JAP) and reverse transcribed using poly (A) polymerase with a Mir-X miRNA First-Strand Synthesis Kit (TAKAR, JAP), following the manufacturer’s instructions. The cDNA was amplified and quantified using TB Green® Premix Ex Taq™ II (Tli RNaseH Plus) Kit on BIO-RAD PCR system (Biorad, USA).The amplification reactions were as follows: 95 °C for 30 s, 40 cycles of 95 °C for 5 s and 60 °C for 30 s, and dissociation at 60 °C for 1 s and 95 °C for 1 s. U6 snRNA was served as an internal control. The miRNA-specific 5′ primers used in the qPCR experiments are shown in Additional file 8: Table S7. Each sample was tested in triplicate. The 2^−△△Ct^ method was used to calculate relative expression.

### Statistical analysis

The data were analyzed by GraphPad Prism7 software. The experimental data are expressed as the mean ± standard deviation (x ® ± s).

## Supplementary Information

**Additional file 1: Table S1.** The details of small-RNA sequencing information and subsequent data analysis.

**Additional file 2: Table S2.** The number of miRNA in all samples.

**Additional file 3: Table S3.** Additional table.

**Additional file 4: Table S4.** Additional table.

**Additional file 5: Fig. S1**. KEGG analysis of exclusively miRNAs in W and F. The bubble chart shows enriched differentially expressed genes in signaling pathways. (A) Bubble chart of the top 20 pathways in exclusively miRNAs in W. (B) Bubble chart of the top 20 pathways in exclusively miRNAs in F. The Y-axis label represents the pathway and the X-axis label represents the rich factor (rich factor = amount of differentially expressed genes enriched in the pathway/amount of all genes in background gene set). The color and size of the bubble represent enrichment significance and the amount of differentially expressed genes enriched in the pathway, respectively. W represents the wild tree shrews, and F represents the domestic tree shrews.

**Additional file 6: Table S5.** Additional table.

**Additional file 7: Table S6.** Additional table.

**Additional file 8: Table S7.** The miRNA-specific 5′ primers used in the qPCR experiments

## Data Availability

All data generated and analyzed during this study are included in this articles as figures and supplemental files.

## Abbreviations

BP: Biological Process
CC: Cellular Componen
DE: Differentially Expressed
GO: Gene Ontology
HCV: Hepatitis C virus
HBV: Hepatitis B virus
KEGG: Kyoto Encyclopedia of Genes and Genomes
MF: Molecular Function
