# Supplementary material for: Comparing the hippocampal miRNA expression profiles of wild and domesticated Chinese tree shrews (Tupaia belangeri chinensis)
Source: BMC Ecol Evol. 2021 Jan 25;21:12. doi: 10.1186/s12862-020-01740-2 (PMC7853310; doi:10.1186/s12862-020-01740-2)
Supplement: Supplementary file 1 — Additional file 1: Table S1. The details of small-RNA sequencing information and subsequent data analysis. [file 12862_2020_1740_MOESM1_ESM.docx]

**Table S1**

The details of small-RNA sequencing information and subsequent data analysis.

| Sample | Clean reads | High-quality | Reads mapped to known *Tupaia belangeri* genome |
| --- | --- | --- | --- |
| W1 | 12,285,640 | 12,127,859 | 7,581,303 |
| W2 | 11,857,538 | 11,697,402 | 7,397,118 |
| F1 | 10,559,041 | 10,415,806 | 6,480,290 |
| F2 | 10,442,413 | 10,296,956 | 6,465,604 |
| F3 | 10,062,894 | 9,928,461 | 6,444,011 |
| F4 | 11,519,597 | 11,291,252 | 7,000,361 |
