# Supplementary material for: Comparing the hippocampal miRNA expression profiles of wild and domesticated Chinese tree shrews (Tupaia belangeri chinensis)
Source: BMC Ecol Evol. 2021 Jan 25;21:12. doi: 10.1186/s12862-020-01740-2 (PMC7853310; doi:10.1186/s12862-020-01740-2)
Supplement: Supplementary file 2 — Additional file 2: Table S2. The number of miRNA in all samples. [file 12862_2020_1740_MOESM2_ESM.docx]

**Table S2**

The number of miRNA in all samples.

| Sample | Existing-miRNA | Known-miRNA | Novel-miRNA |
| --- | --- | --- | --- |
| W1 | 158 | 609 | 1115 |
| W2 | 159 | 587 | 1066 |
| F1 | 157 | 604 | 1223 |
| F2 | 158 | 584 | 1119 |
| F3 | 155 | 573 | 1038 |
| F4 | 156 | 601 | 860 |
