# Supplementary figures and images for: Comparing the hippocampal miRNA expression profiles of wild and domesticated Chinese tree shrews (Tupaia belangeri chinensis)
Source: BMC Ecol Evol. 2021 Jan 25;21:12. doi: 10.1186/s12862-020-01740-2 (PMC7853310; doi:10.1186/s12862-020-01740-2)

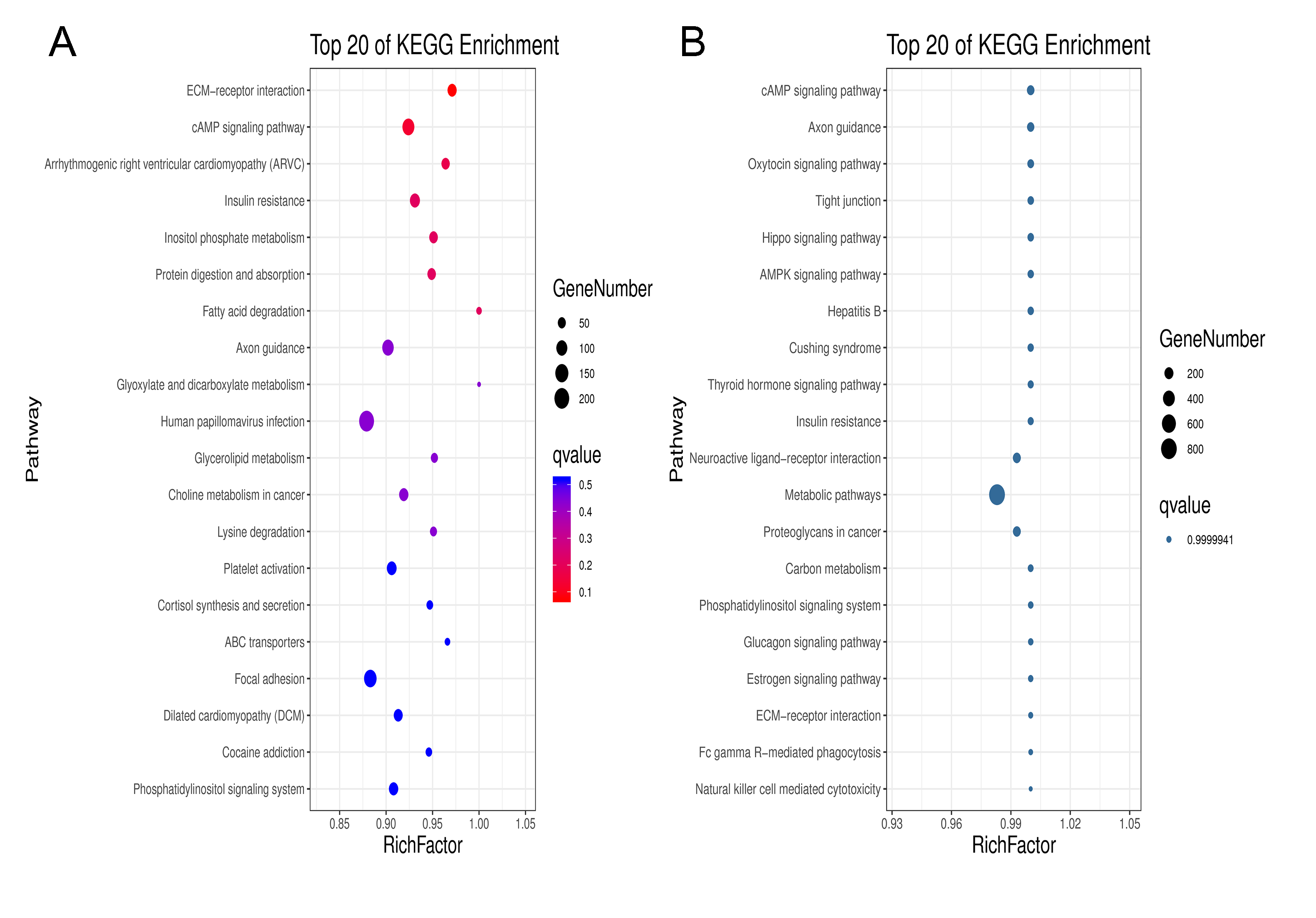

Supplement: Supplementary file 5 — Additional file 5: Fig. S1. KEGG analysis of exclusively miRNAs in W and F. The bubble chart shows enriched differentially expressed genes in signaling pathways. (A) Bubble chart of the top 20 pathways in exclusively miRNAs in W. (B) Bubble chart of the top 20 pathways in exclusively miRNAs in F. The Y-axis label represents the pathway and the X-axis label represents the rich factor (rich factor = amount of differentially expressed genes enriched in the pathway/amount of all genes in background gene set). The color and size of the bubble represent enrichment significance and the amount of differentially expressed genes enriched in the pathway, respectively. W represents the wild tree shrews, and F represents the domestic tree shrews. [file 12862_2020_1740_MOESM5_ESM.tif]
