# Supplementary material for: Comparing the hippocampal miRNA expression profiles of wild and domesticated Chinese tree shrews (Tupaia belangeri chinensis)
Source: BMC Ecol Evol. 2021 Jan 25;21:12. doi: 10.1186/s12862-020-01740-2 (PMC7853310; doi:10.1186/s12862-020-01740-2)
Supplement: Supplementary file 8 — Additional file 8: Table S7. The miRNA-specific 5′ primers used in the qPCR experiments [file 12862_2020_1740_MOESM8_ESM.docx]

**Table S7**

The miRNA-specific 5’ primers used in the qPCR experiments

| miRNA names | miRNA-specific 5’ primers |
| --- | --- |
| novel-m0746-5p | AAAGCACTCTTTTCTCCATAAGA |
| novel-m1388-5p | TGGCCTGGAGGTCCTGAGACT |
| U6-F | TB Green® Premix Ex Taq™ II (Tli RNaseH Plus) Kit supply |
| U6-R |  |
